# Introduction to the National Cancer Imaging Translational Accelerator (NCITA): a UK-wide infrastructure for multicentre clinical translation of cancer imaging biomarkers

**DOI:** 10.1038/s41416-021-01497-5

**Published:** 2021-07-27

**Authors:** M. A. McAteer, J. P. B. O’Connor, D. M. Koh, H. Y. Leung, S. J. Doran, M. Jauregui-Osoro, N. Muirhead, C. Brew-Graves, E. R. Plummer, E. Sala, T. Ng, E. O. Aboagye, G. S. Higgins, S. Punwani

**Affiliations:** 1 Department of Oncology, University of Oxford, Oxford, UK; 2 Division of Cancer Sciences, The University of Manchester, Manchester, UK; 3 Division of Radiotherapy and Imaging, The Institute of Cancer Research and Royal Marsden Hospital, London, UK; 4 Beatson Institute for Cancer Research, University of Glasgow, Glasgow, UK; 5 Department of Surgery and Cancer, Imperial College London, London, UK; 6 Centre for Medical Imaging, University College London, London, UK; 7 Northern Institute for Cancer Care, Freeman Hospital and Newcastle University, Newcastle upon Tyne, UK; 8 Department of Radiology, University of Cambridge and CRUK Cambridge Centre, Cambridge, UK; 9 UCL Cancer Institute, University College London, London, UK; 10 School of Cancer and Pharmaceutical Sciences, King’s College London, London, UK

**Keywords:** Cancer imaging, Cancer imaging

## Abstract

The National Cancer Imaging Translational Accelerator (NCITA) is creating a UK national coordinated infrastructure for accelerated translation of imaging biomarkers for clinical use. Through the development of standardised protocols, data integration tools and ongoing training programmes, NCITA provides a unique scalable infrastructure for imaging biomarker qualification using multicentre clinical studies.

Imaging biomarkers have potential to provide objective, minimally invasive tools for the diagnosis and therapeutic management of tumours, including quantitative assessment of tissue microstructure, metabolism and function. However, clinical imaging within healthcare systems remains largely limited to assessment of anatomical changes in tumour size, with a few notable exceptions such as assessment of tumour metabolism using positron emission tomography (PET) with 2-deoxy-2-[fluorine-18]fluoro-D-glucose integrated with computed tomography ([^18^F]FDG PET-CT) [1, 2]. This gap between translatable imaging biomarker research and clinical application reflects the challenges in crossing two key ‘translational gaps’ [3, 4]; namely translation of promising candidates into fully validated and standardised products and the provision of robust evidence to support their implementation and adoption into clinical practice [5]. For complex multicentre imaging biomarker studies, differences in imaging sequences for different scanners, field strengths and vendors and inter- and intra-observer variability in image acquisition and analysis pose significant challenges for standardisation. Here, we outline a new coordinated infrastructure designed to accelerate the clinical translation of robust and reproducible imaging biomarkers to transform cancer diagnosis and improve healthcare outcomes for patients.

The National Cancer Imaging Translational Accelerator (NCITA) is a clinical imaging research consortium, which is creating the necessary integrated infrastructure to accelerate robust qualification of cancer imaging biomarkers for clinical use, as proposed in the Imaging Biomarker Roadmap for Cancer Studies [5]. Established in 2019, through Cancer Research UK (CRUK) Accelerator award funding, NCITA builds on previous Cancer Imaging Centres Initiatives (2008–2018), funded by CRUK and the Engineering and Physical Sciences Research Council (EPSRC), which were pivotal in driving innovation in biomarker research using novel imaging techniques [6–11]. NCITA brings together nine UK centres of excellence for medical imaging, namely, University College London, The University of Manchester, University of Oxford, King’s College London, The Institute of Cancer Research (ICR), Imperial College London, University of Cambridge, University of Glasgow and Newcastle University (Supplementary 1). This multi-institutional collaborative network includes medical imaging experts, clinical oncologists, physicists and research scientists who are developing standardised imaging biomarker qualification pipelines to accelerate the development of qualified imaging biomarkers using multicentre studies.

The NCITA infrastructure includes three cross-institutional units (imaging clinical trials unit (CTU), quality assurance/quality control (QA/QC) and repository units) and three activity groups (engagement, training and contracts management), which together with the Governance Group, work in synergy to facilitate cross-sector translational imaging biomarker research. The goals of NCITA are to establish standardised protocols and locked-down quality-assured processes for imaging biomarker qualification and a federated research data repository for secure data storage and sharing between study sites. The repository incorporates automated QA and post-hoc computational image analysis using containerised workflows, novel machine learning and artificial intelligence (AI) tools (Fig. 1). To improve study efficiencies, NCITA is developing unified contracts, policies and standard operating procedures that transcend individual institutions. Through engagement with key imaging stakeholders, NCITA will develop consensus guidelines for robust qualification of imaging biomarkers to instill reliability in the development pathway and accelerate clinical adoption. NCITA also organises ongoing workshops, events and online training materials to ensure that the valuable skills and expertise of the NCITA workforce are sustainable and scalable.

**Fig. 1:**
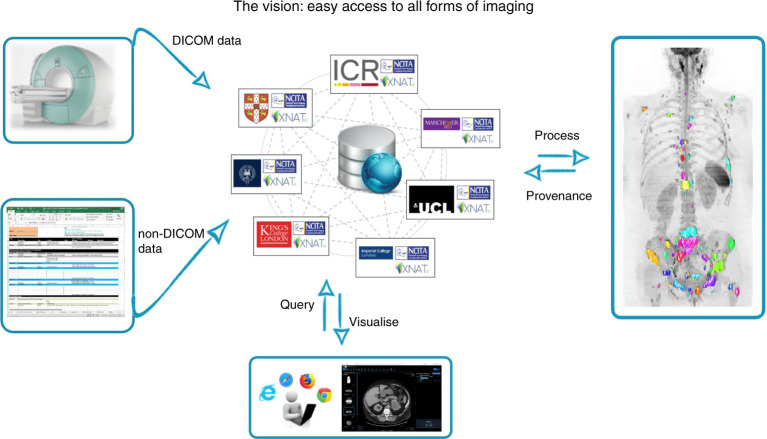
High-level schematic overview of the NCITA Image Repository Unit. Clinical study data from both DICOM and non-DICOM modalities are curated securely, enabling users to perform both automated data quality assurance (thus improving data harmonisation) and bespoke analysis using containerised workflows, including novel AI tools. Processing outputs will be stored back in the repository with complete provenance. Cohort-building tools, which may use natural language processing or content-based image retrieval, together with enhanced visualisation, will allow clinicians and scientists to participate in virtual tumour boards, to enable collaborative knowledge sharing and communication for improving clinical interpretation of complex imaging biomarker readouts.

NCITA supports a portfolio of Exemplar studies, selected to demonstrate the effectiveness of the infrastructure in accelerating qualification and translation of these promising imaging biomarkers (Supplemental Table). NCITA infrastructure support is also accessible to external clinical researchers from academia and industry through NCITA’s study adoption process. International partners are also eligible to apply. For example, NCITA provides repository support for CRUK International Alliance for Cancer Early Detection (ACED), which includes partners at Stanford University and Oregon Health and Science University, USA. Researchers are encouraged to contact NCITA early during study development by email (ncita.general@ucl.ac.uk) or via the website (ncita.org.uk/contact), preferably prior to applying for grant funding. While cancer imaging studies are a key focus, clinical research studies in other disease areas involving AI algorithm development, training and validation will be considered, especially if these studies are thought to benefit AI algorithm development for future early cancer detection studies. Study applications are reviewed by the programme and unit managers and the governance group to assess the quality of the study proposal, funding plans and status (Supplementary 2). Where studies are successfully adopted, a representative from the new institution or company will be encouraged to join the relevant NCITA management, trial steering committee or consensus group to facilitate knowledge transfer and potential growth of the required infrastructure.

Early phase observational and validation-based studies as well as later phase interventional and therapeutic studies are supported by the NCITA CTU using a range of imaging technologies, including whole-body MRI, hyperpolarised ^13^C MRI, multi-parametric MRI, oxygen-enhanced MRI, PET, PET-MRI and PET-CT. The NCITA CTU complements local study infrastructure and provides an adaptable governance framework that can be tailored to the study development phase. This bespoke service provides excellent value for money and aids smoother funding applications and approvals. This is especially important for observational imaging studies, which are often deprioritised when competing for limited National Institute for Health Research (NIHR) infrastructure support, in favour of interventional cancer studies.

The QA/QC Unit supports study setup, technical and biological validation, protocol development and staged lockdown of standardised image acquisition, processing and analysis methodologies. Efficient management of site equipment, process documentation, workflows, audits, training and continuous quality improvements across study sites is facilitated by a centralised quality management system. The QA/QC Unit is also establishing a national MRI Core Lab, to support site evaluation, site qualification and performance monitoring for MRI multicentre studies (Supplementary 3). This will help to elevate the quality of MRI biomarkers and improve translation efficiency. For NCITA PET imaging studies, NCITA will liaise closely with the UK PET Core Lab at St Thomas’ Hospital London to ensure the studies are performed to agreed standards.

The Repository Unit is developing a sustainable, cross-institutional federated repository structure for secure data storage, integration, analysis and sharing. The repository is based on an eXtensible Neuroimaging Archive Toolkit (XNAT) prototype, originally built and hosted by the ICR [12] under the aegis of ICR’s CRUK-EPSRC Cancer Imaging Centre, and related work at the CRUK Cambridge Institute. The repository supports short- or long-term secure data archival, and encompasses the full range of data maturity including initial data acquisition, through various levels of data curation, to controlled release of deidentified data as a community resource. For example, the NCITA repository already contains a large COVID database including the UK National Covid-19 Chest Imaging Database (NCCID). The repository will be interoperable with and receive data directly from the National Health Service (NHS), including NHS Digital’s Trusted Research Environment services.

NCITA’s data sharing solutions are based on the FAIR principles [13], that data are findable, accessible, interoperable and reusable. For NCITA’s adopted studies, streamlined data access procedures will enable data sharing, including an approach to the relevant Ethics and Trial Steering Committees. For non-NCITA sites, data access will involve an application process and will be regulated by audited procedures. Open-access data resources will also be developed for the imaging research community worldwide.

The Repository and QA/QC Units are developing quality-assured pipelines for imaging biomarker qualification from source image data. Recent work includes implementation of the VERDICT technique [14], radiomics pipelines in line with International Biomarker Standardisation Initiative guidelines [15] and a plugin to enable automatic assessment of incoming imaging datasets to determine compliance with study protocols. The Repository Unit is also developing AI-assisted image annotation for radiologists, linking of curated imaging datasets to publications and generation of datasets for AI development. The Repository Unit is also engaged in the release lifecycle of XNAT and is the integrator of the Open Health Imaging Foundation (OHIF) viewer into XNAT [16], which provides a flexible framework for building web-based imaging applications to support clinical imaging research.

A key goal for NCITA is to generate consensus on how the imaging biomarker development pathway can be improved to achieve more efficient translation of quality-assured imaging biomarkers into clinical practice. The NCITA Engagement Group are engaging with key imaging stakeholders, to facilitate the establishment of a consensus group to agree and publish recommendations for accelerating robust qualification and adoption of imaging biomarkers for clinical use. The Engagement Group also facilitates clinical researchers from academia and industry to access the NCITA infrastructure and works closely with CRUK’s Commercial Partnerships team to ensure translational support for new discoveries arising from exemplar projects and other projects supported by CRUK. Other engagement activities include dissemination of news, events and information through the NCITA website (https://www.ncita.org.uk), social media accounts (NCITA Imaging (@imaging_cancer)|Twitter and LinkedIn), press releases and publications.

During the 5-year CRUK Accelerator Award, NCITA will have established the necessary integrated infrastructure to facilitate accelerated translation of imaging biomarkers for clinical application. To ensure continued growth and sustainability, NCITA will link with NHS initiatives including the NIHR Biomedical Research Council/Clinical Research Network and NIHR Imaging Group, as well as funding bodies, charities and industrial partnerships. The sustainability of NCITA will have significant benefits in streamlining future clinical imaging studies and providing ongoing training of a new generation of highly skilled medical imaging researchers to accelerate personalised and precision medicine for cancer care.

## Supplementary information

Supplemental Material

## Data Availability

Not applicable.
