# Supplementary material for: Introduction to the National Cancer Imaging Translational Accelerator (NCITA): a UK-wide infrastructure for multicentre clinical translation of cancer imaging biomarkers
Source: Br J Cancer. 2021 Jul 27;125(11):1462–5. doi: 10.1038/s41416-021-01497-5 (PMC8313668; doi:10.1038/s41416-021-01497-5)
Supplement: Supplementary file 1 — Supplemental Material [file 41416_2021_1497_MOESM1_ESM.pdf]

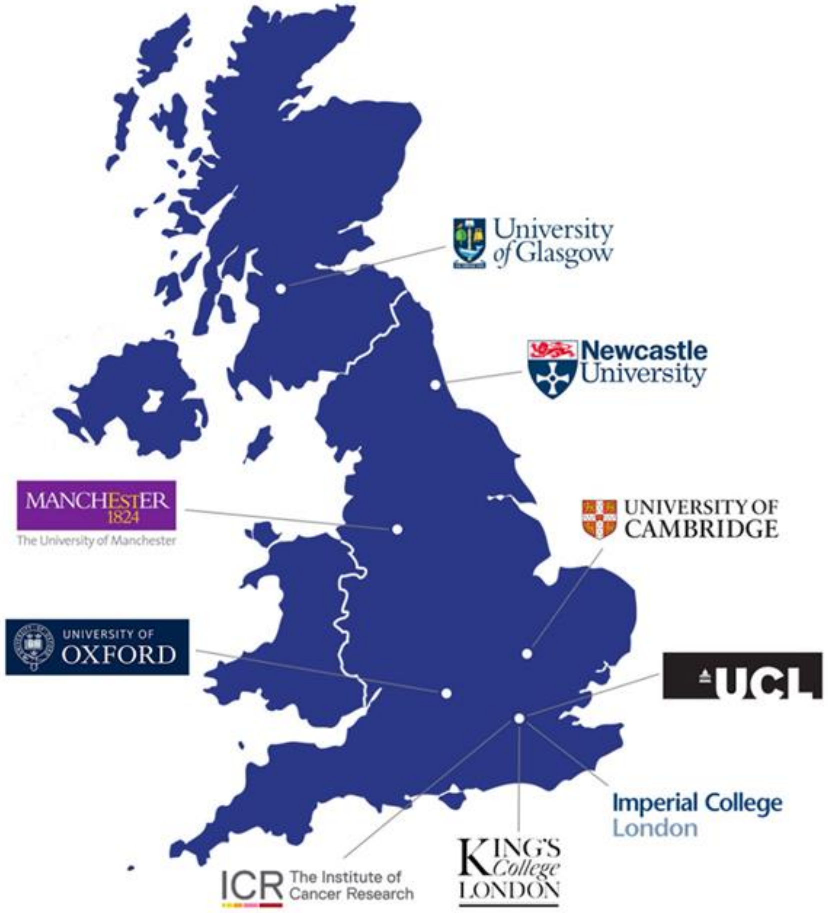

Supplementary 1

Supplemental Table

Summary of NCITA Exemplar Studies

Exemplar Study	**Scope**
**[^{18}F]FPIA PET/CT imaging in patients with solid tumours.**	Multicentre feasibility study to assess the association between [^{18}F]FPIA PET imaging of short chain fatty acid uptake in solid tumours and tumour proliferation.
**MISSION-Fumarate Trial**	Validation of hyperpolarised [1,4-$^{13}C_2$]fumarate as a candidate prognostic and treatment response marker for renal cell carcinoma.
**MUK Nine b Trial**	Establishment of the environment for UK multicentre clinical evaluation of whole-body MRI as a diagnostic and treatment response marker in multiple myeloma.
**Novel Image Repository for multi-parametric MRI prostate cancer trials**	Development of a novel multi-parametric MRI prostate image repository using data from the PROMIS, PRECISION, INNOVATE and RE-Imagine clinical trials including the development of artificial intelligence automated reporting and multicentre clinical trial.
**The Foam Study**	Multicentre phase 1 study to establish the multi-platform Feasibility of Oxygen enhAnced MRI for adaptive radiotherapy planning in non-small cell lung cancer.
**EXODIMER-PET**	Exosome analysis of HER2 expression and heterodimerisation in patients from the HERPET study at Imperial College London.
**The FIG Trial**	Multicentre phase 1 study to assess the feasibility of performing [^{18}F]FDOPA PET guided histopathology using standardised PET imaging protocols.
**PANORAMA Trial**	Interventional, non-randomised, multicentre study to evaluate the feasibility of PSMA [^{68}Ga]Ga-PSMA PET/CT imaging as a tool to guide treatment choice in patients with high risk prostate cancer.

Abbreviations

[^{18}F]FPIA [^{18}F]fluoropivalic acid; [^{18}F]FDOPA 3,4-dihydroxy-6-[^{18}F]fluoro-L-phenylalanine; HER2 human epidermal growth factor 2; PSMA Prostate-specific membrane antigen; Ga gallium.

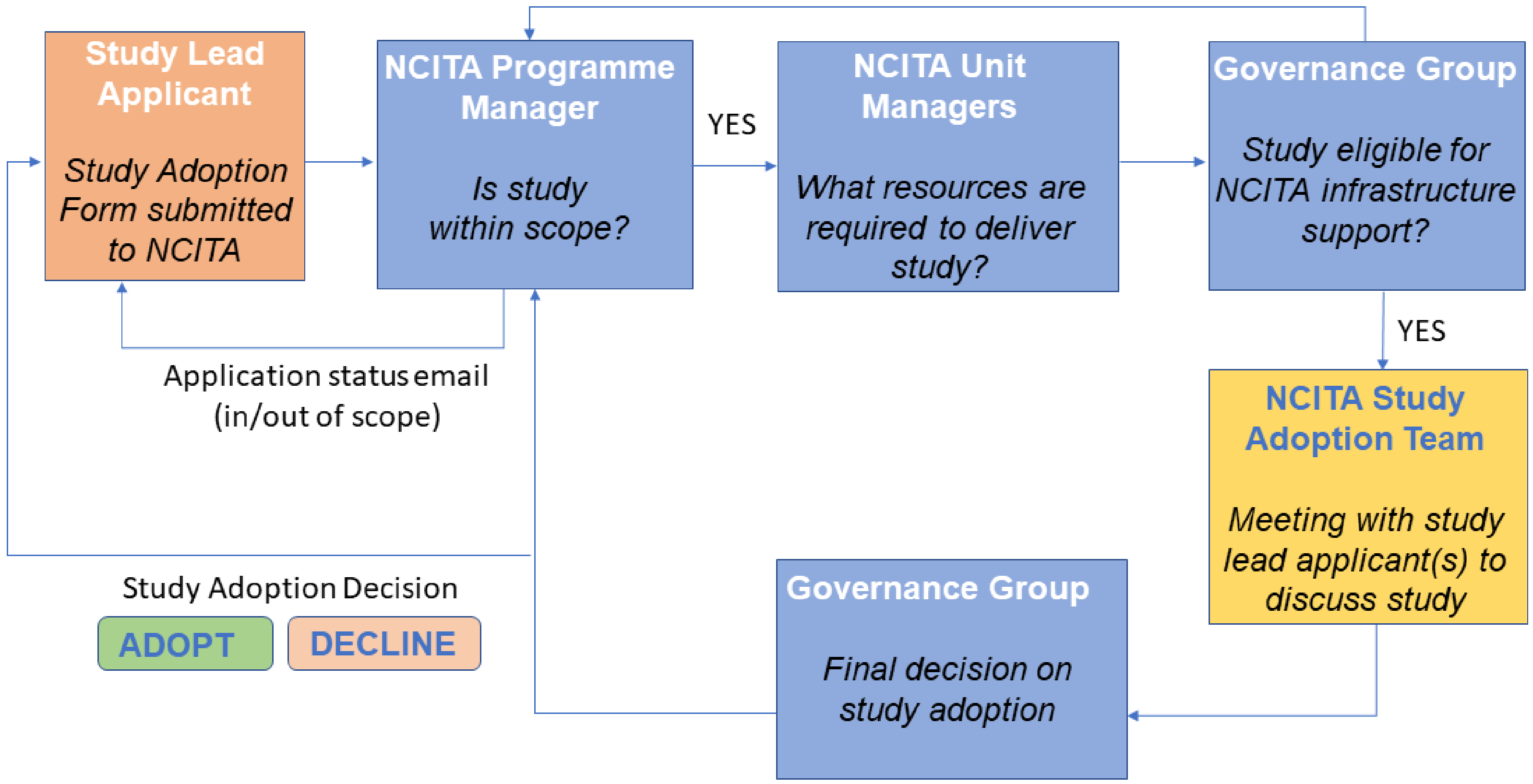

Supplementary 2

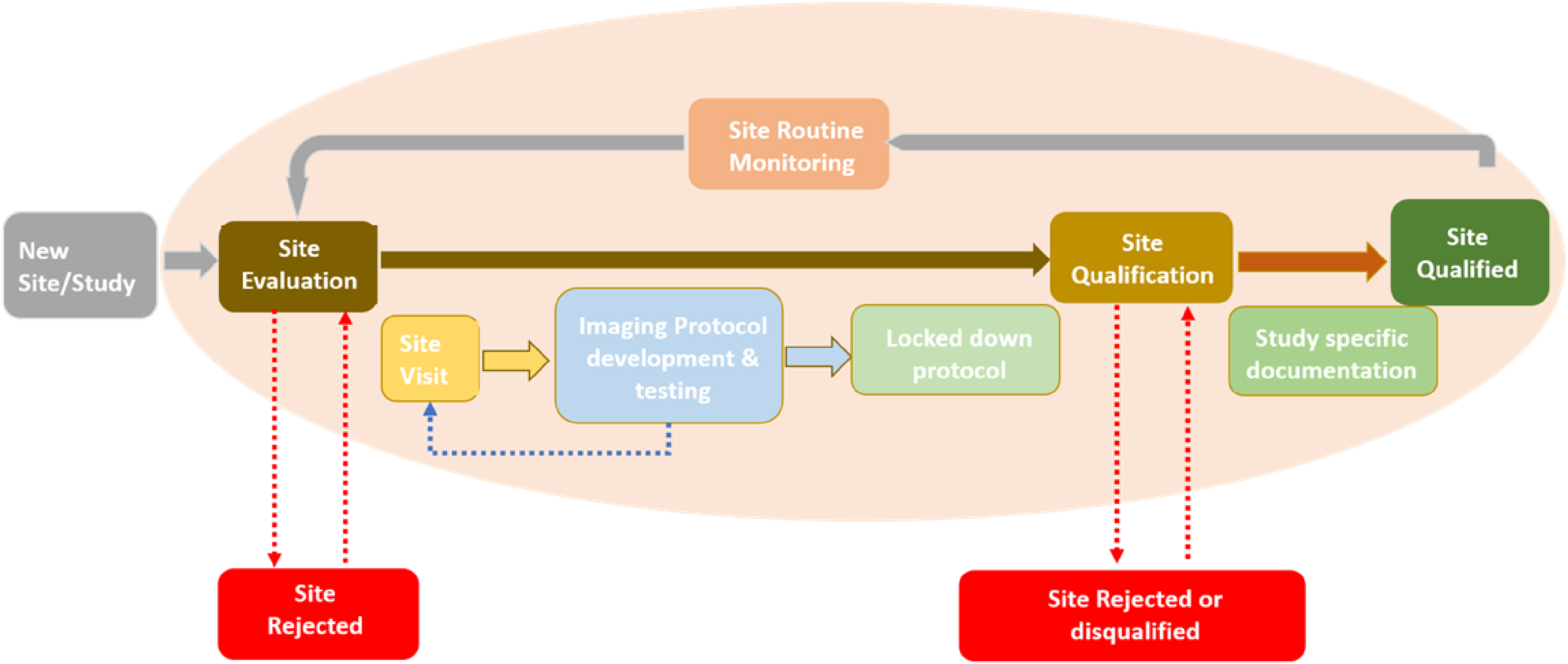

Supplementary 3

Supplementary 1 NCITA partners across the UK

NCITA brings together nine academic partners (Cambridge, Glasgow, Imperial College London, Institute of Cancer Research London, King's College London, Manchester, Newcastle, Oxford University College London) whose expertise spans MRI and nuclear imaging research, radiology, data repository management, image analysis and statistical support. The academic institutions work in partnership with regional NHS Hospital Trusts to facilitate the delivery of multicentre clinical imaging research studies.

Supplementary 2 NCITA Study Adoption Process

NCITA study applications made to ncita.general@ucl.ac.uk are rapidly triaged by the NCITA programme manager and relevant unit managers. Subsequent review by the Governance Group according to the NCITA study eligibility criteria will lead to adoption decision within 30 days. Studies formally adopted are monitored by the relevant NCITA unit(s) against defined study protocols and agreements, with NCITA support acknowledged on adopted study publications and presentations.

Supplementary 3 Site evaluation and qualification by the NCITA QA/QC MRI Core Lab

The NCITA QA/QC Unit provides independent site evaluation for new imaging biomarker studies and staged lockdown of imaging study protocols to ensure high-quality study design, image data acquisition, processing and analysis methodologies at the qualified study sites to improve the sensitivity and accuracy of imaging biomarkers for clinical use.
